# Supplementary material for: One model fits all: Combining inference and simulation of gene regulatory networks
Source: PLoS Comput Biol. 2023 Mar 27;19(3):e1010962. doi: 10.1371/journal.pcbi.1010962 (PMC10079230; doi:10.1371/journal.pcbi.1010962)

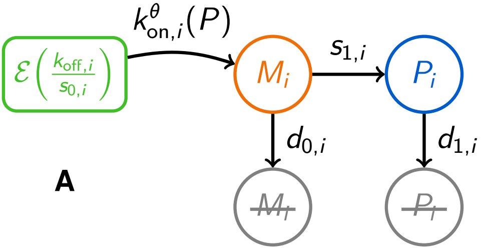

B

$$\begin{cases} M_i(t) \xrightarrow{k_{\mathrm{on},i}^{\theta}(P(t))} M_i(t) + \mathcal{E}\left(\frac{k_{\mathrm{off},i}}{s_{0,i}}\right) \\ M_i'(t) = -d_{0,i} M_i(t) \\ P_i'(t) = s_{1,i} M_i(t) - d_{1,i} P_i(t) \end{cases}$$

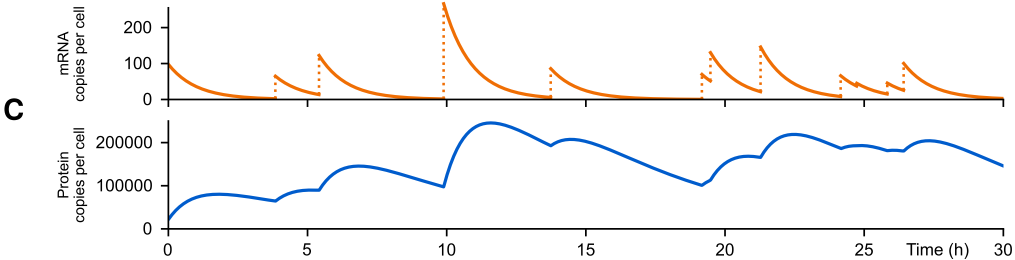

Supplement: S1 Fig — Related to Fig 1. Graphical (A) and mathematical (B) descriptions of the mechanistic model for the dynamics of a gene i. (C) Bursts of mRNA occur at random times with rate kon,i and their size follows an exponential distribution E(koff,i/s0,i). The variables Mi and Pi describe respectively the mRNA and protein quantities associated to gene i in the cell. The vector of protein levels is denoted by P = (P1, ⋯, Pn) while θ denotes the GRN which couples the genes together through functions kon,i. (PDF) [file pcbi.1010962.s003.pdf]
