# Supplementary figures and images for: One model fits all: Combining inference and simulation of gene regulatory networks
Source: PLoS Comput Biol. 2023 Mar 27;19(3):e1010962. doi: 10.1371/journal.pcbi.1010962 (PMC10079230; doi:10.1371/journal.pcbi.1010962)

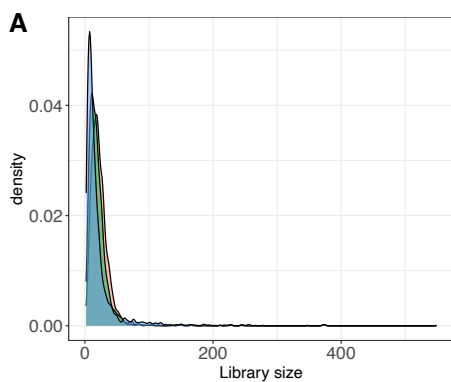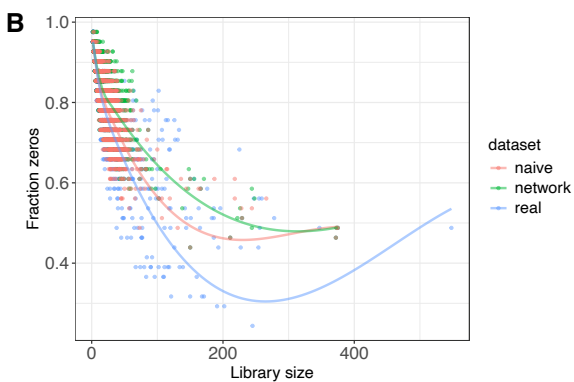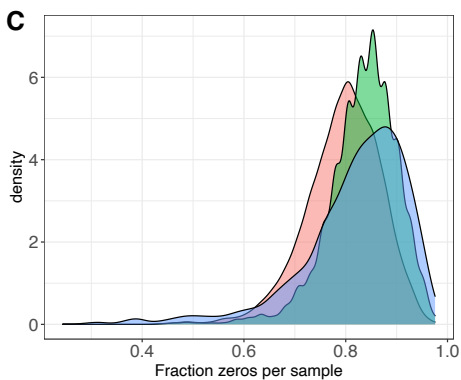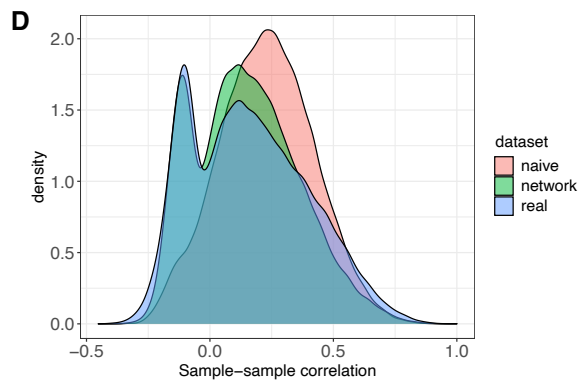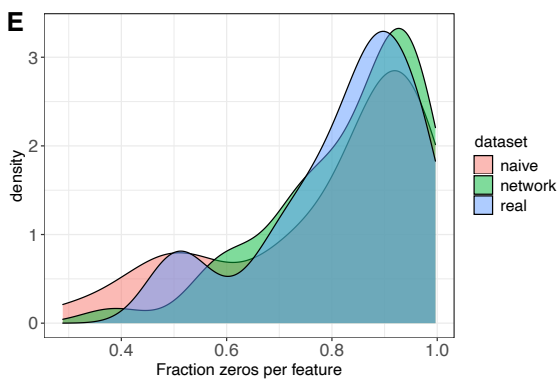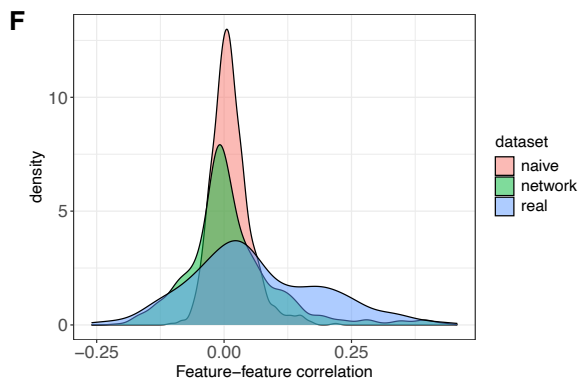

Supplement: S2 Fig — Related to Fig 7. Comparison of various statistical characteristics across datasets using the countsimQC package. Each plot shows the experimental dataset (real, in blue) and datasets simulated from the mechanistic model calibrated by CARDAMOM, including interactions (network, in green) and without interactions (naive, in red). Each dataset consists of 41 genes (features) measured in 2433 single cells (samples). (A) Distribution of “library sizes”, defined as the total read count in each sample. (B) Association between the library size and the fraction of zeros observed per cell. (C) Distribution of the fraction of zeros observed per cell. (D) Distribution of cell-cell correlations, based on random cell pairings. (E) Distribution of the fraction of zeros observed per gene. (F) Distribution of gene-gene correlations, based on all possible gene pairings. Notably, the cell-cell correlation (D) bimodal pattern shows two possible pairings of cells: pairs with similar expression profiles (same genes on, same genes off) and therefore positively correlated, and pairs with opposite, “antinomic” profiles and therefore negatively correlated. This pattern is an indirect sign of the emergence of different cell types, a characteristic that is clearly not reproduced in the absence of interactions between genes (naive dataset). (PDF) [file pcbi.1010962.s004.pdf]

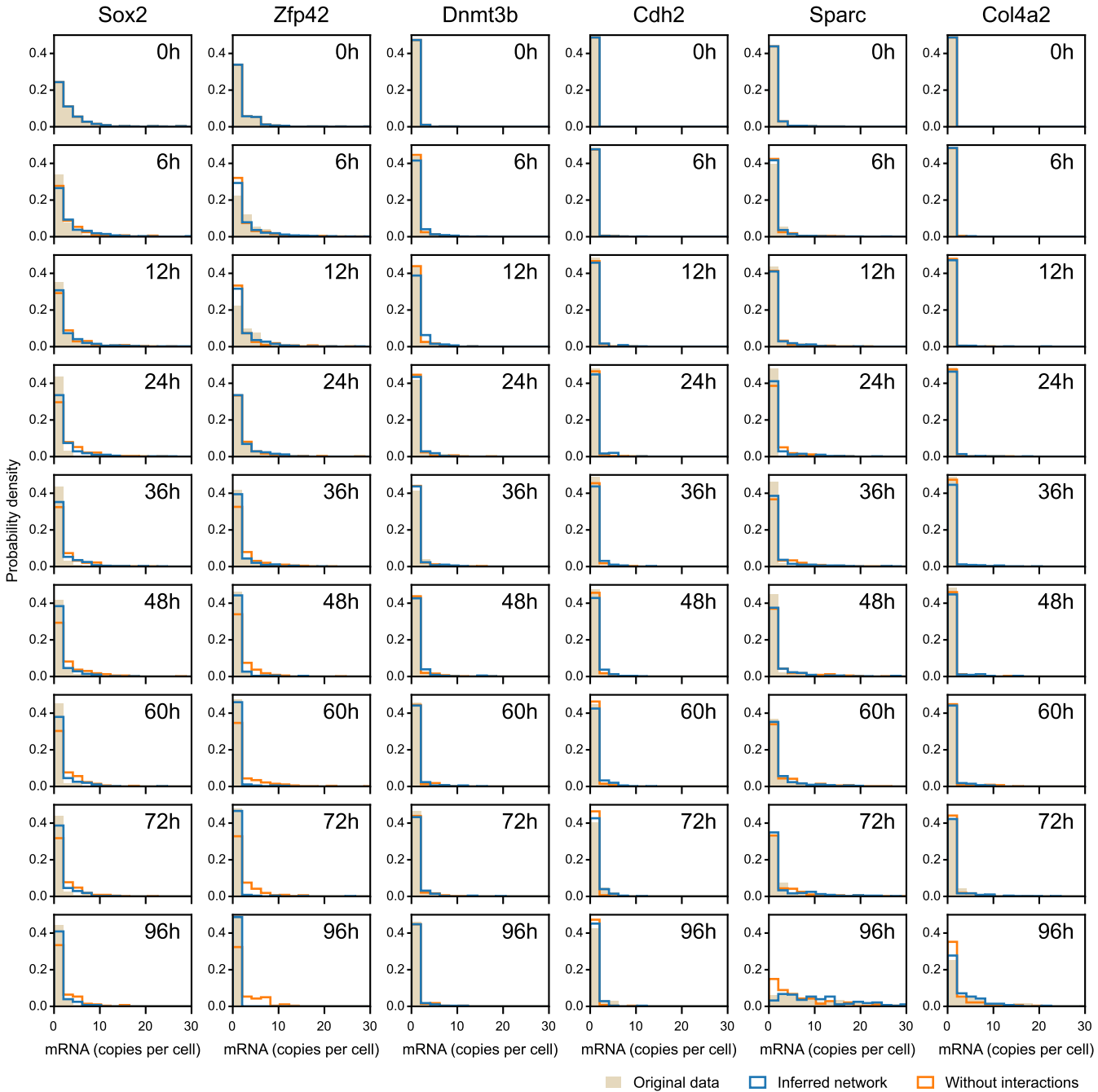

Supplement: S3 Fig — Related to Fig 7. Comparison between empirical distributions along timepoints, for six genes that have been found to play a key role in the regulation of the process (as visible in Fig 5). The experimental dataset (in beige), the dataset simulated from the inferred network (in blue) and the dataset simulated without interactions (in orange) correspond to Fig 7E, 7F and 7G, respectively. (PDF) [file pcbi.1010962.s005.pdf]

**A** Original data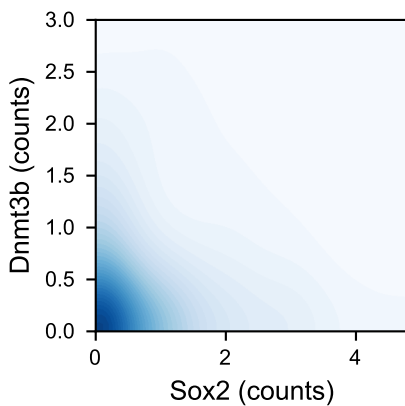**B** Inferred network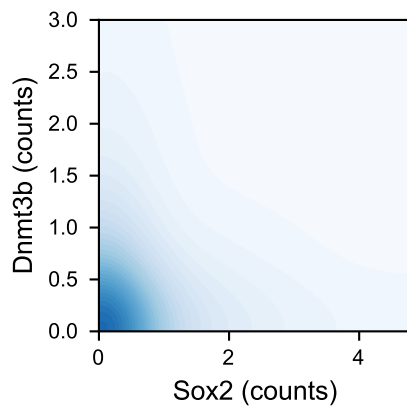**C** Without interactions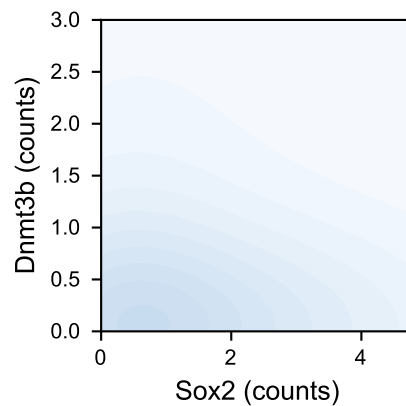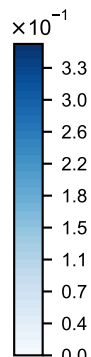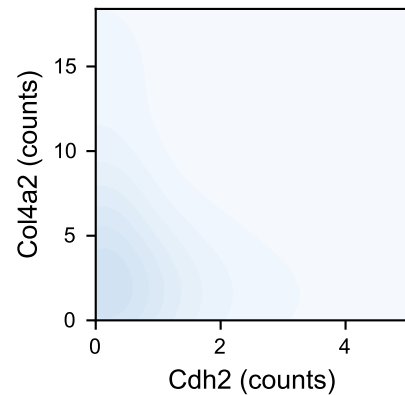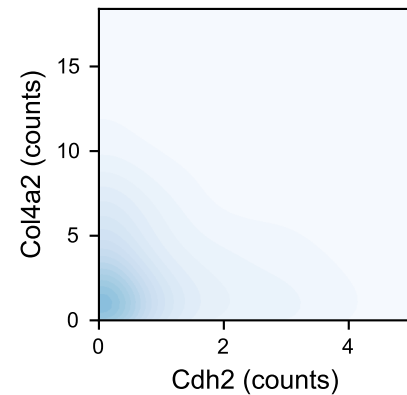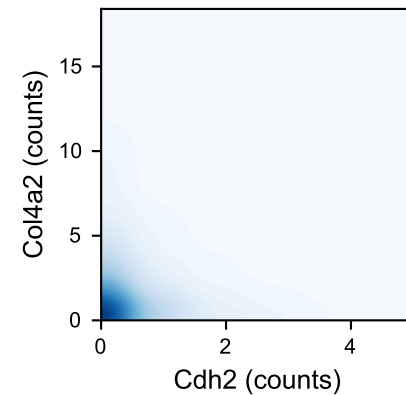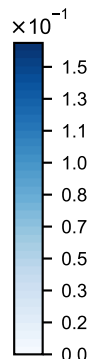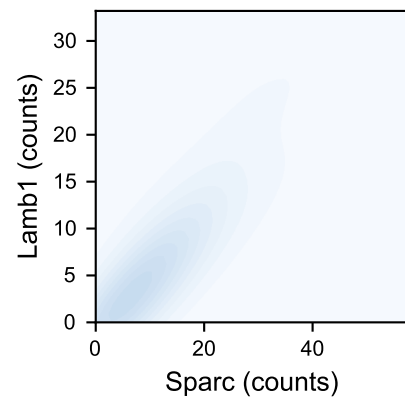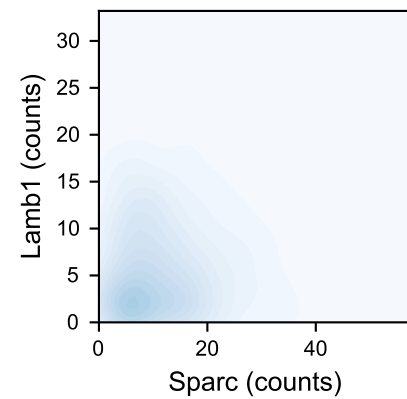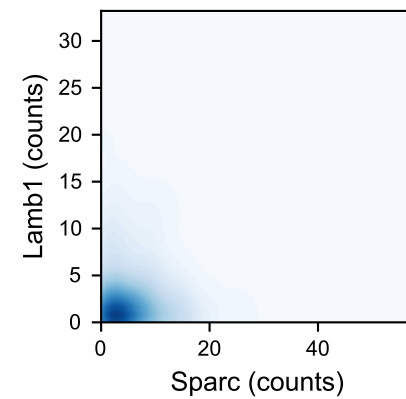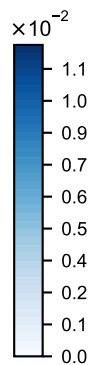

Supplement: S4 Fig — Related to Fig 7. Comparison of the joint distributions of three pairs of genes at the final timepoint between the experimental dataset (A) compared to the dataset simulated when the mechanistic model is calibrated by CARDAMOM (B) and the dataset simulated without interactions (C). The genes in each pair are expected to have interactions (direct or indirect) in the network represented in Fig 5. (PDF) [file pcbi.1010962.s006.pdf]

KS test p-values

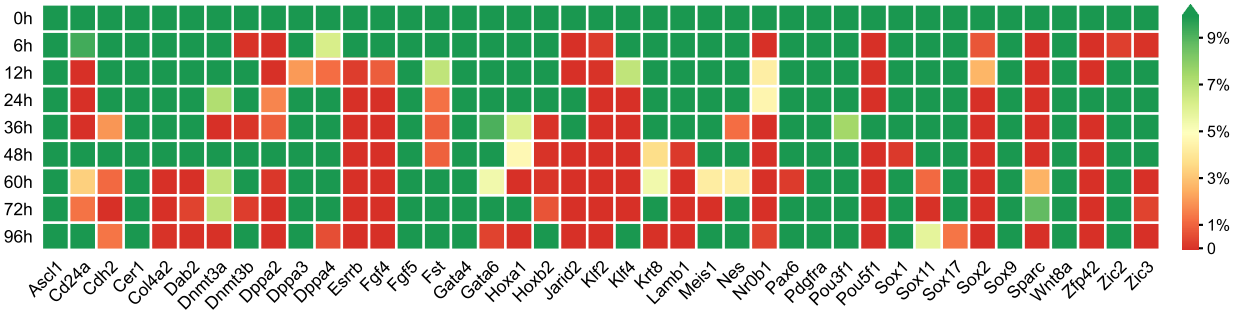

Supplement: S6 Fig — Related to Fig 7. Heatmap of p-values associated with Kolmogorov–Smirnov (KS) tests between real mRNA distributions and the ones simulated from the null network (without interactions), for each of the 41 genes of the network and for each timepoint. The green color indicates p-values greater than 5%, implying that the model output is not significantly different from the experimental dataset. (PDF) [file pcbi.1010962.s008.pdf]

Pluripotency

Extraembryonic endoderm

Post-implantation epiblast

Neuroectoderm

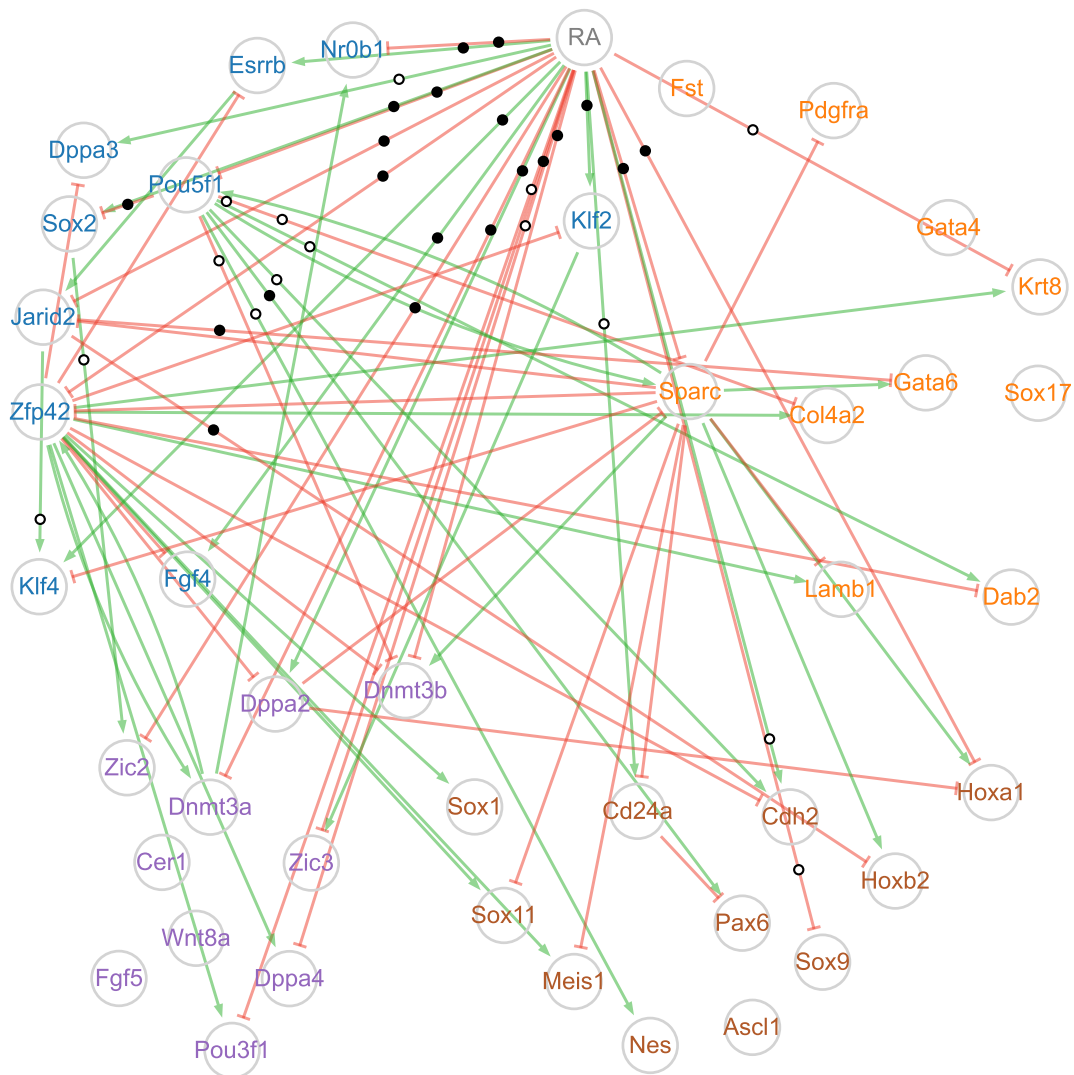

Supplement: S7 Fig — Related to Fig 5. The SINCERITIES inference method was applied to the experimental dataset from [22] restricted to a panel of 41 marker genes identified by the authors. The network structure is obtained by keeping only the 5% strongest activations (green arrows) and inhibitions (red blunt arrows) acting on each gene. Genes are colored according to four groups related to different cell states (pluripotency, post-implantation epiblast, neuroectoderm, extraembryonic endoderm) following the proposed classification of [22]. Edges supported by a ChIP-seq interaction are marked with black dots (see main text for the definition of what is considered as an interaction) and edges that are not supported are marked with white dots: this concerns only the edges starting from the RA stimulus, Pou5f1, Sox2, and Jarid2. Edges for which we have no reliable information have no mark. (PDF) [file pcbi.1010962.s009.pdf]
