# Supplementary material for: One model fits all: Combining inference and simulation of gene regulatory networks
Source: PLoS Comput Biol. 2023 Mar 27;19(3):e1010962. doi: 10.1371/journal.pcbi.1010962 (PMC10079230; doi:10.1371/journal.pcbi.1010962)
Supplement: S5 Fig — Related to Fig 7. Two-dimensional UMAP representations of the experimental dataset (original data) and datasets simulated after calibrating the mechanistic model with CARDAMOM, one including interactions (inferred network) and one obtained after setting θ = 0 (without interactions). The four plots are based on two different projections, computed after merging the experimental dataset with (A-B) the simulated dataset including interactions or (C-D) the simulated dataset without interactions. Hence A and C are two representations of the exact same data, while B is to be compared with A, and D is to be compared with C. (PDF) [file pcbi.1010962.s007.pdf]

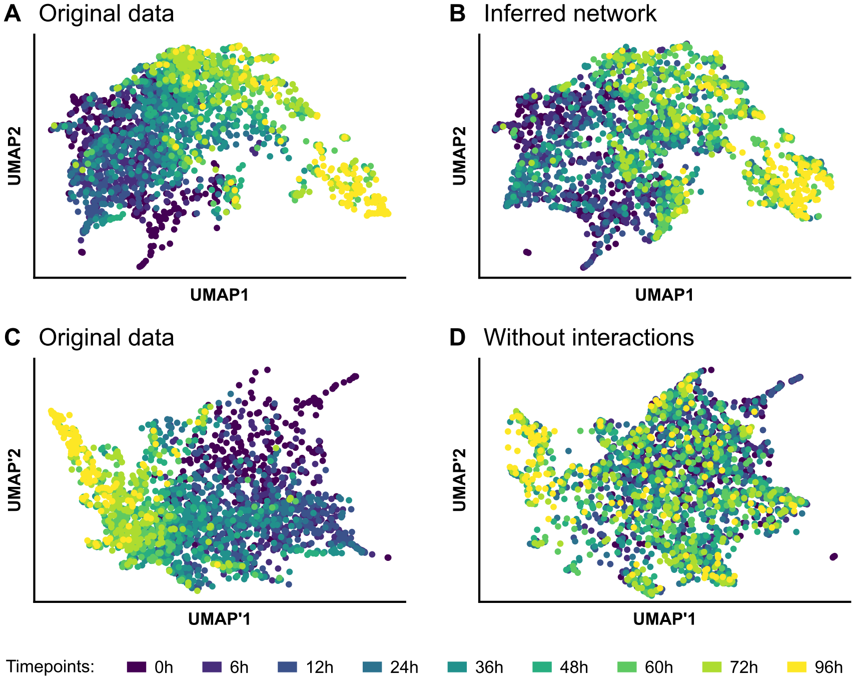
A Original data
UMAP2
UMAP1
B Inferred network
UMAP2
UMAP1
C Original data
UMAP'2
UMAP'1
D Without interactions
UMAP'2
UMAP'1
Timepoints: 0h 6h 12h 24h 36h 48h 60h 72h 96h
