# Supplementary material for: One model fits all: Combining inference and simulation of gene regulatory networks
Source: PLoS Comput Biol. 2023 Mar 27;19(3):e1010962. doi: 10.1371/journal.pcbi.1010962 (PMC10079230; doi:10.1371/journal.pcbi.1010962)
Supplement: Attachment — Submitted filename: Report.pdf [file pcbi.1010962.s010.pdf]

Report on the manuscript PCOMPBIOL-D-22-01102 entitled
One model fits all: combining inference and simulation of gene regulatory networks

This paper presents a strategy to infer a gene regulatory network (GRN) from time course transcriptomic single cell data, and also simulate with this model data trajectories that present "realistic" transcriptional profiles. It relies on two algorithms proposed in previous works by the authors, Harissa and Cardamom, based on a mechanistic model that takes into account the transcriptional bursting process (codes available on a github). The validation of the method relies on different steps. First, *in silico* data are generated (trajectories of mRNA and proteins) with the algorithm Harissa, a python package for inferring gene regulatory networks from single-cell data. Nine different datasets corresponding to five different graph structures have been generated. A sampling of cells at different time points is done to obtain time stamped snapshot. Then, the authors realised a benchmark testing 6 different inference methods, among them Harissa and Cardamom. Finally, they consider a real data set, and use Cardamon, the algorithm providing the best performance in their benchmark tests, to first infer a GRN model, and then simulate data profiles and compare them with experimental data and a "null network" model.

The paper is well written and addresses current interesting and difficult challenges the inference of network model from time stamped data, and the simulation of data trajectories that satisfy main expected features.

Here are some remarks and questions:

- The authors talk about GRN, an interaction graph, and also about GRN model, a GRN with parameters that provide the dynamics caused by the interactions. In the literature, most of the methods presented infer GRNs, but there are also a few that infer GRN models. This distinction does not stand out well in the manuscript, and it is sometimes a bit confusing...

  The sentence lines 79-82 in the introduction is not so clear : what is a phenomenological model? What means "gene expression patterns, and especially transitions between cell types, are hard-coded"?

- Cardamon needs time stamped data instead of keeping the temporary ordered cells. Can this pre-processing step add some difficulty in the comparison with real single cell data trajectories ?

- The "best" inference method in this benchmark (Cardamom) is built on the same mathematical model as the one used to generate the data (Harissa). Is there not a bias that will contribute to the good performance of the inference...?

- Although Cardamon generally dominates the other methods tested, performance seems to depend on the type of graph. When we don't know

a priori which type of graph is underlying, why do not test several (two) methods on the real data and compare? Cardamon is well fitted in case of transcriptional bursting, but what happens if some regulations of the GRN are less concerned by this phenomena...?

- The third result (from line 234) could be organised differently, starting with a description of the inferred GRN, then its annotation and analysis... (it is just a suggestion, not mandatory !).

- Although a time dependence of degradation rates was observed in the data (and is commented on in the discussion), the decision to multiply by a scaling factor of 6 at time 72h seems arbitrary and clearly has an impact on the result. Did the factor of 6 and the time chosen come from the observations, or were they compared for validation?

- Looking at the p-values of the Sparc and Esrrb genes when compared with the experimental data set: could the poor results be related to their very specific role in the GRN (one is a strongly inhibited output; the other a hub controlling a large part of the graph)? Other nodes with low p-value have a quite high degree (Sox2, Dnmt3a...). Possible link with local properties of the node ?

- The first part of the title "one model fits all" is not so clear... all what?

Questions concerning the simulations

- Harissa is used to generate data. The incidence matrix $\theta_{i,j}$ is given by the desired structure of the graph, but are there other parameters to fit, and to which values are they set ?

- l 153: what is a "condition"? Is it the type of the graph?

- Lines 186-196: It is not clear on which data sets has been really tested SCRIBE

- To reproduce in vitro experiments, the model is first running without stimulus, until the steady state is reached. Then, the reached steady state is used as the initial condition for the simulations with the stimulus at 1. For some model (as FN4), there are several steady states when the stimulus is off. Here, for the model FN4, it seems (Figure 1) that the state 0000 is the initial state. Did you try the others? Sensitivity to the initial condition?

- To measure the algorithms performance, the AUPR curve is used, but without taking account the diagonal terms in a sake of simplicity (as explained in Methods). However, in networks type like FN4 and FN8, the self regulation do play a major role in the dynamical features (differentiation)? In these cases, is their deletion not questionable?

Minor corrections

- line 110: Remove "type of" (the 9 datasets correspond to 5 types of datasets, and the Tree-type has been declined in 5 sizes)
- What is the meaning of the dashed gray line in Figure 3 (AUPR=0,2)
- The x-axis of Figure 3C is not so natural compared to the legend... (density of measurements)
- Figure 5: should precise in the legend that the annotation (black and white dots) is done only for the edges directly linked to RA
- line 273 : 85% (not 0.85%)
- References for Harissa and Cardamom differ along the text (between 6, 15, 23...)
- Figure S4 : No color code
- the words cell/sample and characteristic/gene are used without any difference... sometimes disturbing to have two words for the same concept, especially when they are close in the text)
