# Supplementary material for: One model fits all: Combining inference and simulation of gene regulatory networks
Source: PLoS Comput Biol. 2023 Mar 27;19(3):e1010962. doi: 10.1371/journal.pcbi.1010962 (PMC10079230; doi:10.1371/journal.pcbi.1010962)
Supplement: Attachment — Submitted filename: response_letter.pdf [file pcbi.1010962.s011.pdf]

Response to review comments

We would like to thank the reviewers for carefully reading our manuscript and helping us to improve the quality of the paper. In the revised version of this manuscript, all modifications made following suggestions appear in red. Below is a point-by-point response to questions and comments of the reviewers.

Reviewer 1

No modification asked.

Reviewer 2

1. *I would have appreciated to have a more explicit introduction to the technicalities of the different tools used and a more clear identification of the differences to previous publications on these two tools.*

   We precisely tried to not enter into too much details in the technicalities of the different tools and refer to the previously published papers of the two methods since "In this work, we sought to investigate the benefits of using this model as an integrated tool for both GRN inference and data simulation [...] therefore assessed its ability to allow for efficient network reconstruction from time-course scRNA-seq data, while accurately reproducing the dataset main features from the functioning of the inferred network" (line 84). We believe that this achievement deserves this independent article, accessible to the larger community of systems biologists, free of the technical considerations behind these algorithms.

   Nevertheless, we have expanded the paragraph entitled "Tested algorithms" in the Methods section, where we briefly describe CARDAMOM and HARISSA, as well as the one entitled "Calibration of the mechanistic model". There are few differences from previous publications regarding these tools, although it is worth mentioning that both algorithms have been slightly improved to make them more efficient and compatible with each other. Those differences are described in the file `cardamom_vignette.pdf` on the associated Git repository.

Reviewer 3

1. *The authors should clarify whether they used every pair of genes not present in the ground truth as a negative example to compute the number of false positive and true negatives. In this context, I am puzzled as to how the random classifier can have a precision of 0.47 in Figure 4B. This high value suggests a ground truth network where nearly half the possible edges are present.*

   Importantly, we only have a ground truth (which we recall is not absolute; it just ensures the existence of a physical contact between proteins of the source gene and the promoter region of the target gene, but not necessarily an effect) for the interactions from four particular nodes of the GRN (the RA stimulus, Pou5f1, Sox2 and Jarid2, as stated in the legend of Figure 4) so we use only these interactions (4×41 edges) to compute the number of false negatives and false positives. It turns out that the four nodes are highly connected to the other genes according to this ground truth, explaining the high score of the random classifier (indeed nearly half of the 164 edges are present). Following the reviewer's comment on code availability, the true network and the inferred networks are

now available online (Git repository of CARDAMOM): we hope that it will help to clarify this point for those who may be surprised by the high score of the random classifier.

2. *The authors should include a supplement that studies the network inferred by GENIE3 (or SINCERITIES) in a manner similar to page 11 and Figure 5.*

   This is now added in supplementary (Figure S7). We chose SINCERITIES because it is the only algorithm, apart from CARDAMOM and HARISSA, that infers both interactions and their sign (inhibition or activation), making the comparison with the network inferred by CARDAMOM easier. We have added a related paragraph in Results at line 330.

3. *In Figure 7A, the p-values should be corrected for testing multiple hypotheses. I am not sure if they are. The authors could reduce the number of tests by performing one K-S test for each gene considering its expression over all time points. It will also be instructive to see such a plot for the naive model without interactions.*

   The plot for the naive model without interactions has been added in the supplementary (Figure S6). Besides, we have added at line 380:

   "This observation is confirmed by smaller p-values (i.e., significant discrepancies from the experimental data) for many more genes and timepoints when removing interactions between genes (Figure S6)."

   Regarding the multiple testing correction, it is important to note that Figure 7A is somehow the *opposite* of a multiple testing situation. Indeed, the multiple testing situation would correspond to claiming a "success" (positive) for each red box (low p-value), hence the need to control the false positive rate. But here it is the contrary: the more tests we do, the more demanding we are about the quality of the model, and the goal is to have "as few red boxes as possible". Thus, applying a correction (which is roughly equivalent to *increasing* all p-values) here would mean being *less demanding* with the quality of the model and not the other way round: this is why we have not applied such a correction in this figure.

4. *On page 3, lines 79-80, the authors state that "existing GRN-based simulation tools, which are generally based on more phenomenological than mechanistic models". It may be that my understanding of "phenomenological" and "mechanistic" is different, but isn't BoolODE based on simulating a Boolean (mechanistic) model rather than a phenomenological one? In Discussion, the authors state that SERGIO uses a mechanistic model.*

   Indeed we had not mentioned BoolODE, which is probably with SERGIO one of the few GRN simulation models that is closest to what we call mechanistic in this paper. We call 'mechanistic' a model for which *cell behavior is an emergent property of the hypotheses made to build the model*, so a 'mechanistic GRN model' is a model for which *cell behavior is an emergent property of the underlying GRN.* In BoolODE, the drift is built in a mechanistic way from the Boolean network that represents the interactions, making the deterministic behavior of the cell indeed really emerging from the network. However, in this model, the variability is added in a second step through a non-biological Brownian noise term: in that respect, the noise is not mechanistic according to our definition. Importantly, the noise generated by standard models of transcriptional bursting turns out to be very different from Brownian noise, at least at the mRNA level.

   Regarding SERGIO, we explained in the paper that if SERGIO indeed uses a mechanistic model, part of the cell behavior is "hard-coded" in the model through the activity of specific "master" transcription factors. Thus the switch between different cell types does not emerge from the network. Moreover, the noise in this model is built *a posteriori* so

that the variability matches that observed in experimental data: the variability is not mechanistic either.

Based on this point and the first point of Reviewer 4, we have added a new paragraph in Introduction at line 49. We have also added an explicit reference to BoolODE in the discussion, together with SERGIO, in the paragraph starting line 424.

5. *page 18, line 447: "by going up the arrow of time" may be better phrased as "by going back in time".*

   Corrected (now line 480).

6. *page 21, line 558: Change "bursts" to "burst".*

   Corrected (now line 594).

7. *Several citations are missing page numbers.*

   Corrected.

8. *page 21, line 561: The meaning of the phrase "a synthetic noise well adapted" is unclear. Perhaps this sentence should be rephrased.*

   Corrected (now line 595).

Code availability

- *The software for both HARISSA and CARDAMOM are available but I do not see the network files for the toy networks in Figure 1, the simulated data for the corresponding analyses, or the ground truth networks for Figure 4. The authors should provide code to generate all the results in their manuscript, even if in the form of Jupyter notebooks.*

  These files have been added to the CARDAMOM repository (`https://github.com/eliasventre/cardamom`), which now contains code to generate all the results.

  In particular, we have added a directory called `results_article` which contains itself two directories. `Benchmark_on_simulated_data` contains all the scripts to generate the simulated data, with explicit names. `Semrau_network_analysis` contains the inferred networks and `build_real.py` allows to create the ChiP-seq based reference network known from the RA stimulus and the three genes Pou5f1, Sox2 and Jarid2 to the other genes.

Reviewer 4

1. *The authors talk about GRN, an interaction graph, and also about GRN model, a GRN with parameters that provide the dynamics caused by the interactions. In the literature, most of the methods presented infer GRNs, but there are also a few that infer GRN models. This distinction does not stand out well in the manuscript, and it is sometimes a bit confusing... The sentence lines 79-82 in the introduction is not so clear : what is a phenomenological model? What means "gene expression patterns, and especially transitions between cell types, are hard-coded"?*

   As the reviewer points out, there are three levels that we distinguish: (1) the graph level, where the GRN denotes only interactions without any description of the dynamics; (2) the phenomenological level, where the GRN is related to a model of gene expression, but such that the cell behavior (cell types, gene expression variability) is not biologically arising from the model; (3) the mechanistic level, where such behavior is an emerging property of the model (i.e., not directly encoded by some dedicated “external” parameters). Following

the reviewer's question and point 4 of Reviewer 3, we have added a paragraph at line 49, for this distinction to be clearer and for defining what we call "mechanistic model" throughout the article; we also refer to the answer addressed to point 4 of Reviewer 3.

2. *Cardamon needs time stamped data instead of keeping the temporary ordered cells. Can this pre-processing step add some difficulty in the comparison with real single cell data trajectories ?*

   Importantly, CARDAMOM does not require any pre-processing step in our framework. More precisely, except for SCRIBE, all the methods considered here are snapshot-based and thus do not require pre-processing: "Crucially, they do not require the observation of cell trajectories, whose inference is a problem in itself" (line 73). On the other hand, SCRIBE is a trajectory-based method and thus it does need a pre-processing step. As shown in Figure 2C, this pre-processing step turns out to be very unreliable in presence of transcriptional bursting. Indeed, SCRIBE performs well given exact trajectories (in brown, corresponding to a "perfect" idealized pre-processing and unavailable experimentally) but it has poor results given the same data as the other methods (light green and pink): the limiting factor here is not SCRIBE itself but the trajectory-reconstruction algorithms, which make too many errors because of bursty gene expression.

   So the "difficulty" in our framework does not come from data pre-processing, but rather data acquisition, since one has to sample cells over time-course experiments. This type of data is obviously a minority at the moment (often we have access to a set of samples in no particular order), but there are already at least a dozen published datasets of this type (for example ref. 22, 24 and 25), and we advocate continuing in this direction (as observed at line 239, having many timepoints is at least as important as having many cells).

3. *The "best" inference method in this benchmark (Cardamom) is built on the same mathematical model as the one used to generate the data (Harissa). Is there not a bias that will contribute to the good performance of the inference...?*

   Yes, this is an important point that the benchmark section aims to "verify that CARDAMOM quantitatively reconstructs causal links when the data is simulated from HARISSA" (abstract) and to "verify that the two model-based methods perform better than the others on these datasets" (line 96). The comparison with other algorithms aims to show that the inference is not trivial for this model, and is also an opportunity to test their efficiency in case of transcriptional bursting. However, we emphasize that one of the major achievements of our article is not to outperform the other algorithms, but to efficiently reverse-engineer the mechanistic GRN model while the interactions are not trivial to infer from the data: the sentence at line 160 has been expanded to reflect this. Note that this bias does not stand when using a real dataset, where CARDAMOM performance is still demonstrably better (Figure 4A-B).

4. *Although Cardamom generally dominates the other methods tested, performance seems to depend on the type of graph. When we don't know a priori which type of graph is underlying, why do not test several (two) methods on the real data and compare? Cardamon is well fitted in case of transcriptional bursting, but what happens if some regulations of the GRN are less concerned by this phenomena...?*

   Indeed, performance of CARDAMOM depends on the type of graph. However, CARDAMOM and HARISSA are the only two methods allowing to simulate the model after the inference, as they provide together with the interactions the sign and the intensity in adequacy with the burst rate functions k_{on}. Thus, the purpose of the benchmark is to verify that the network is correctly inferred for any type of graphs, but for simulation purposes

we have not the choice between the different methods, even when they outperform ours (for the network CN5, in particular).

The second point of the question is more subtle: CARDAMOM is well fitted in case of transcriptional bursting, whatever the size of the bursts. Thus, if some regulations of the GRN are less concerned by the phenomenon, it will simply correspond to a case where some genes i have a burst size $s_i/k_{\text{off},i}$ that is low, and maybe as well a low mRNA degradation rate d_i. This would be detected by the first step of the inference method and should not affect the performances. Nevertheless, in that case, it is probable that the other methods would be more competitive, as they are significantly affected by transcriptional bursting while CARDAMOM and HARISSA are specifically designed for taking it into account.

5. *The third result (from line 234) could be organised differently, starting with a description of the inferred GRN, then its annotation and analysis... (it is just a suggestion, not mandatory !).*

   This was precisely what was envisaged in a first version of the manuscript, but we finally decided to present the section in this form to simplify the sequence of ideas.

6. *Although a time dependence of degradation rates was observed in the data (and is commented on in the discussion), the decision to multiply by a scaling factor of 6 at time 72h seems arbitrary and clearly has an impact on the result. Did the factor of 6 and the time chosen come from the observations, or were they compared for validation?*

   The multiplication of the synthesis and degradation rates at t=72h (equivalent to the multiplication of the last timepoint) comes from the observation of the data. It has of course a huge impact on the results since most of the genes of the Extraembryonic endoderm group would have not reached the expected state at t=96h without this modification. We observed that the experimental distributions are well reproduced by the model as soon as it reaches steady state at t=96h, which turns out to correspond to a multiplicative factor greater than ≈ 6. Thus, we could arbitrarily choose any factor greater than 6 without changing the result, since the model would have reached the stationary distribution associated to the inferred network. On the other hand, a smaller factor would lead to incomplete dynamics as the final state would not have been reached. We have added some clarification in the Methods section at line 691.

7. *Looking at the p-values of the Sparc and Esrrb genes when compared with the experimental data set: could the poor results be related to their very specific role in the GRN (one is a strongly inhibited output; the other a hub controlling a large part of the graph)? Other nodes with low pvalue have a quite high degree (Sox2, Dnmt3a...). Possible link with local properties of the node ?*

   This is a very good remark: indeed, we observe a lower p-value for Sparc, Sox2, Dnmt3a, which are genes with a high connectivity in the network. However, we believe that their low p-value is not related to the local property of the nodes, but simply to the fact that we observe for these genes a complex behavior during the process, which cannot be precisely described by our simple model. This has been discussed line 464: "if the distribution of a gene is more complex than a mixture of these two modes, the model is not expected to reproduce accurately its dynamics, since minor regulatory interactions might go undetected (especially if a third "hidden" mode is close to one of the two main modes). This seems to be the case for example for the slight decrease of Sparc at t = 24h, which would have been better captured by adding an extra mode."

   The fact that these genes have a complex behavior leads the inference method to detect them as important nodes of the network, because they have to be regulated by potentially

many other genes to explain this behavior, and their variation makes them good candidates for explaining variations of other genes (in particular for Sparc). We have added a paragraph in the discussion at line 471 to detail this.

8. *The first part of the title "one model fits all" is not so clear... all what?*

   The reviewer is right that a more precise title should be "One model fits both" (inference and simulation). We chose this "catchy" title in the hope that it would stimulate the reader's curiosity, making sure that the second part of the title specifies what is meant by "all". As we have not had any comments on this from the other three reviewers, we hope that the reviewer will forgive us for this touch of humor, which we hope will help to give this article the visibility we expect. Importantly, statistical frameworks used for GRN inference are often very different from the GRN models used for data simulation and thus difficult to combine, which the title tends to highlight.

9. *Harissa is used to generate data. The incidence matrix $\theta_{i,j}$ is given by the desired structure of the graph, but are there other parameters to fit, and to which values are they set ?*

   There are indeed other parameters: $k_{0,i}$, $k_{1,i}$, $s_{0,i}/k_{\text{off},i}$, and β_i (introduced in the Methods section). The first three parameters are fitted during the first step of CARDAMOM, directly from the parameters α of the Gamma-mixture fitted to the data, while the parameter β_i is fitted along with θ_{ij} in the second step. We have added a paragraph in the section "Simulation of time-stamped datasets" (line 575), and made some clarifications in the section "Calibration of the mechanistic model" (line 672).

10. *l 153: what is a "condition"? Is it the type of the graph?*

    The term "condition" was used in place of "network", this has been corrected.

11. *Lines 186-196: It is not clear on which data sets has been really tested SCRIBE*

    We have added clarifications (now lines 198-212). The SCRIBE algorithm has been tested on the same datasets as the ones used for the other algorithms, but unlike the others, it is trajectory-based and thus needs a pre-processing step to first reconstruct cell trajectories from the snapshots. We used two different methods for such reconstruction (scenarios 2 and 3). Apart from that, scenario 1 uses a different dataset made of real cell trajectories for comparison purposes, which would correspond to a "perfect" pre-processing step. We have added in the legend of Figure 2: "For the last two conditions, the datasets used are therefore the same as those used for the other methods."

12. *To reproduce in vitro experiments, the model is first running without stimulus, until the steady state is reached. Then, the reached steady state is used as the initial condition for the simulations with the stimulus at 1. For some model (as FN4), there are several steady states when the stimulus is off. Here, for the model FN4, it seems (Figure 1) that the state 0000 is the initial state. Did you try the others? Sensitivity to the initial condition?*

    This is not exactly the case, because our model does not behave as boolean network and depending on the value of the parameters, the number of basins may change. In any case, for generating the data of the benchmark, we ran for every network the model without stimulus during a time $t = 5h$ and took it as an initial condition. For FN4, the value of the basal parameters explain that the initial distribution obtained with this method is very close to be uniformly 0. We chose these basal parameters so that the signal propagation would allow the edges of the network to be active at some point of the measurement, and it seems then possible to infer the full network from the data. A different initial distribution (obtained with different basal parameters) could lead to an unidentifiable problem, and

would then decrease the performance of all methods, making their comparison more difficult. Note that we did not choose basal parameters such that our methods were able to infer the network, but we rather built networks (including basal parameters) such that the model simulations show a clear signal propagation through gene differential expression. It appears that from these data, our methods were able to reconstruct accurate networks.

13. *To measure the algorithms performance, the AUPR curve is used, but without taking account the diagonal terms in a sake of simplicity (as explained in Methods). However, in networks type like FN4 and FN8, the self regulation do play a major role in the dynamical features (differentiation)? In these cases, is their deletion not questionable?*

    This is an interesting point. It is not really for simplicity that we do not take into account the diagonal coefficient, but because "inferring these coefficients is a notoriously difficult task [17]" (line 628). In practice, for most of the algorithms, these diagonal coefficients are detected at a non-negligible level, whether there is a self-regulation or not, as soon as a gene is high at some moment in the dynamical process. In the case of CARDAMOM, the self-regulation is properly detected at a higher level when it is in the network, but also at a smaller level when it does not exist but the gene is activated by another one. The effect is not important when we simulate the model with the inferred network (see Figure 10 in ref. [15]), but it pulls down the AUPR scores because this level is significant compared to the level of other interactions detected. As it is the same for all the other tested algorithms, we decided to remove the diagonal terms for the analysis, to avoid pulling all AUPR scores down, which would have made the comparison less clear. We have added details at line 630 in order to clarify this.

14. *line 110: Remove "type of" (the 9 datasets correspond to 5 types of datasets, and the Tree-type has been declined in 5 sizes)*

    Corrected (now line 119).

15. *What is the meaning of the dashed gray line in Figure 3 (AUPR=0,2)*

    Implicitly same meaning as in Figure 2, but it has been added in the legend of Figure 3.

16. *The x-axis of Figure 3C is not so natural compared to the legend... (density of measurements)*

    This has been corrected.

17. *Figure 5: should precise in the legend that the annotation (black and white dots) is done only for the edges directly linked to RA*

    We have added in the legend: "this concerns only the edges starting from the RA stimulus, Pou5f1, Sox2, and Jarid2."

18. *line 273 : 85% (not 0.85%)*

    Corrected (now line 287).

19. *References for Harissa and Cardamom differ along the text (between 6, 15, 23...)*

    This has been clarified (lines 79, 81, 114, 616). There is one reference for CARDAMOM and two references for HARISSA (ref. 6 presents the full mathematical framework while ref. 23 presents a simplified version and points to the Python implementation). Regarding these two references, which we believe are complementary, the paragraph at line 140 states: "Whereas the original inference module of HARISSA was limited to a few genes [6], it recently integrated an effective CARDAMOM-inspired simplification [23] that allows to infer networks with a much larger number of genes."

20. *Figure S4 : No color code*

    Corrected; the figure has been improved by imposing the same scale for each gene pair.

21. *the words cell/sample and characteristic/gene are used without any difference... sometimes disturbing to have two words for the same concept, especially when they are close in the text)*

    For the words cell/sample, we have replaced “this type of sample normalization” by “this type of normalization” line 652. From a statistical point of view, each cell indeed corresponds to a “sample” (e.g. in Figure S2) since we are working with single-cell data (whereas with bulk data, a sample corresponds to the sum of many cells).

    For the words characteristic/gene, we apologize but we did a search in the text using the word “characteristic” and did not see where it could be confused with the word “gene”. If Reviewer 4 gives us more information about this ambiguity, we could of course change it since these two words must not be used for the same purpose. (Note that in Figure S2 which uses statistical language, each gene is a “feature” but not a “characteristic”.)
